# Exposure of beta-tubulin regions defined by antibodies on an *Arabidopsis thaliana *microtubule protofilament model and in the cells

**DOI:** 10.1186/1471-2229-10-29

**Published:** 2010-02-18

**Authors:** Yaroslav Blume, Alla Yemets, Yarina Sheremet, Alexey Nyporko, Vadym Sulimenko, Tetyana Sulimenko, Pavel Dráber

**Affiliations:** 1 Institute of Food Biotechnology and Genomics, National Academy of Sciences of Ukraine, Kiev 04123, Ukraine; 2 Institute of Molecular Genetics, Academy of Sciences of the Czech Republic, 142 20 Prague, Czech Republic

## Abstract

**Background:**

The function of the cortical microtubules, composed of αβ-tubulin heterodimers, is linked to their organizational state which is subject to spatial and temporal modulation by environmental cues. The role of tubulin posttranslational modifications in these processes is largely unknown. Although antibodies against small tubulin regions represent useful tool for studying molecular configuration of microtubules, data on the exposure of tubulin epitopes on plant microtubules are still limited.

**Results:**

Using homology modeling we have generated an *Arabidopsis thaliana *microtubule protofilament model that served for the prediction of surface exposure of five β-tubulin epitopes as well as tyrosine residues. Peptide scans newly disclosed the position of epitopes detected by antibodies 18D6 (β1-10), TUB2.1 (β426-435) and TU-14 (β436-445). Experimental verification of the results by immunofluorescence microscopy revealed that the exposure of epitopes depended on the mode of fixation. Moreover, homology modeling showed that only tyrosines in the C-terminal region of β-tubulins (behind β425) were exposed on the microtubule external side. Immunofluorescence microscopy revealed tyrosine phosphorylation of microtubules in plant cells, implying that β-tubulins could be one of the targets for tyrosine kinases.

**Conclusions:**

We predicted surface exposure of five β-tubulin epitopes, as well as tyrosine residues, on the surface of *A. thaliana *microtubule protofilament model, and validated the obtained results by immunofluorescence microscopy on cortical microtubules in cells.

The results suggest that prediction of epitope exposure on microtubules by means of homology modeling combined with site-directed antibodies can contribute to a better understanding of the interactions of plant microtubules with associated proteins.

## Background

Microtubules are dynamic cytoskeletal polymers essential for various cell functions such as intracellular organization, ordered vesicle transport, cell division and establishment of cell polarity. In higher plants, several distinct microtubular arrays have been identified, namely the interphase cortical array, preprophase band, mitotic spindle and phragmoplast [1]. The basic building blocks of microtubules are heterodimers of globular α- and β-tubulin subunits. They are arranged in a head-to-tail fashion to form 13 protofilaments that constitute cylindrical microtubules with outer diameter around 25 nm [2]. In *A. thaliana *tubulin subunits are encoded by small gene families, six for α-tubulin [3] and nine for β-tubulin [4]. It has been proposed that the function of microtubules is modulated by highly diverse posttranslational modifications of tubulin dimers [5].

A major advance step in understanding the microtubule function is marked by the solution of its structure, based on docking the high-resolution structure of brain tubulin, studied by electron crystallography [6,7], into lower-resolution microtubule maps imaged by electron cryomicroscopy [8-10]. The microtubular surface displays a surprisingly large number of binding sites, with numerous proteins binding to the outside surface and a multitude of small ligands binding to the inside of microtubules [11]. Some structural interactions with other molecules including nucleotides, drugs, microtubule-associated proteins (MAPs) and motor proteins were predicted [2]. Microtubule models also provide the opportunity to predict the surface location of small antibody epitopes, as well as posttranslationally modified amino acids residues. Antibodies with binding sites on microtubule surface make it possible to study interaction between microtubules and interacting proteins, including tubulin modifying enzymes, in resting cells or cells activated by extracellular stimuli. Site-directed antibodies can also be used for detection of conformation changes in microtubules due to the presence of flexible tubulin domains [12,13]. In spite of a growing number of available anti-tubulin antibodies, data on location of epitopes on native microtubules outside the C-terminal regions of tubulin subunits are very limited. Comparative (homology) modeling makes it possible to predict the structures of proteins with similar sequences [14]; homology modeling of tubulin subunits was used for calculation of discernible differences in tubulin biophysical properties [15] and for a rational design of plant herbicides [16]. However, plant microtubule models were, so far, not reported.

Previously we have found fixation-dependent exposure of tubulin epitopes in *N. tabacum *microtubules [17], and phosphorylation of *N. tabacum *tubulin on tyrosine [18]. However, important questions remained unresolved, namely whether or not cellular microtubules can be phosphorylated and what consequences it might have for microtubular integrity. Furthermore, the function of microtubules in cells responding to extracellular stimuli might be better understood with more knowledge on how the predictions of β-tubulin epitopes and phosphotyrosine locations derived from microtubule model correlate with their exposure in cells.

Here we report on the correlation between localization of small β-tubulin regions on *A. thaliana *microtubule protofilament model and their exposure on cortical microtubules in cells.

## Results

### Epitope mapping

**Figure 1:**
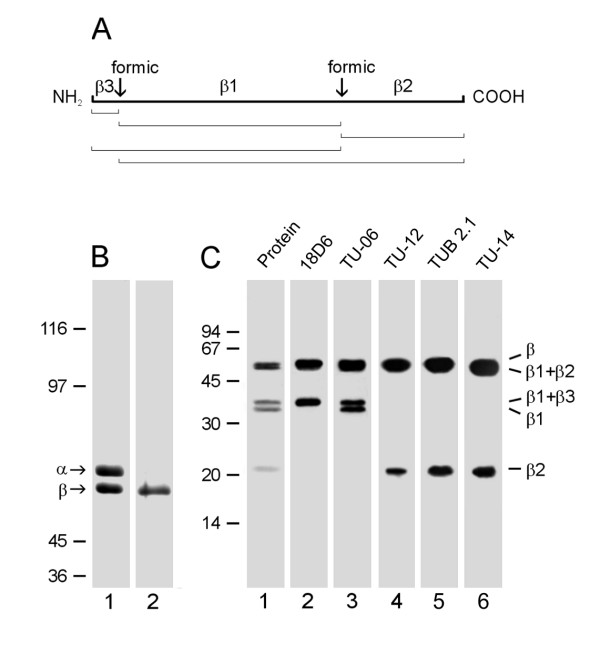
**Reactivity of TU-14 and TUB 2.1 antibodies with β-tubulin fragments**. (A) Schematic representation of the β-tubulin peptide map after formic acid cleavage. The positions of formic acid cleavage sites (formic) and the generated proteolytic fragments (β1, β2 and β3) are shown on the thick line. Fragment sizes after incomplete cleavage are outlined below. (B) Coomassie Blue staining of carboxyamidomethylated tubulin heterodimer (lane 1) and isolated β-tubulin (lane 2). α and β denote positions of tubulin subunits. 7.5% SDS-PAGE. (C) Immunostaining with antibodies to β-tubulin fragments generated by formic acid. Lane 1: protein staining of blotted fragments; lanes 2-6: immunostaining with antibodies 18D6 (marker of β3), TU-06 (marker of β1), TU-12 (marker of β2), TUB 2.1 and TU-14. Positions of β-tubulin fragments are indicated on the right margin. 12.5% SDS-PAGE. Molecular mass markers (in kDa) are indicated on the left of B and C.

Brain tubulin, which can be prepared to a very high degree of purity, is a useful source for epitope mapping. Previous experiments have shown that antibodies TU-14 [19] and TUB 2.1 [20] recognize epitopes in the C-terminal structural domain of porcine brain β-tubulin, while antibody 18.D6 recognizes epitope in the N-terminal region of the molecule [21]. It is known that specific chemical proteolysis (by means of 75% formic acid) of aspartic-proline bonds generates a small number of proteolytic fragments in tubulin dimers [22]. We therefore analysed by immunoblotting porcine brain β-tubulin fragments after formic acid cleavage, using anti-β-tubulin antibodies with known epitope location as markers. β-Tubulin has two aspartic-proline bonds at positions β31-32 and β304-305 (P02554 in the Swiss-Prot Sequence Database) [23]. Formic acid cleavage generates 5 fragments denoted β1, β2, β3, β1+β2 and β1+β3, as outlined in Figure 1A. The subunits were effectively separated by electrophoresis (Figure 1B, lane 1), and the β-tubulin subunit was isolated from gel by electroelution (Figure 1B, lane 2). When purified β-tubulin was subjected to formic acid proteolysis, fragments β1+β3, β1 and β2 were discernible after staining the blotted proteins with SYPRO Ruby Protein Blot Stain (Figure 1C, lane 1). Antibody 18D6, raised against peptide β1-12, served as a marker of the β1+β3 fragment (Figure 1C, lane 2), antibody TU-06 recognizing an epitope in the region β81-95 served as a marker of the β1 fragment (Figure 1C, lane 3), and antibody TU-12 recognizing an epitope in the region β426-435 served as a marker of the β2 fragment (Figure 1C, lane 4). Immunoblotting with TUB 2.1 antibody revealed reactivity with both β2 and β1+β2 fragments (Figure 1C, lane 5). This indicates that the corresponding epitope is located in the region β305-445. Antibody TU-14 also reacted with both β2 and β1+β2 fragments (Figure 1C, lane 6).

**Figure 2:**
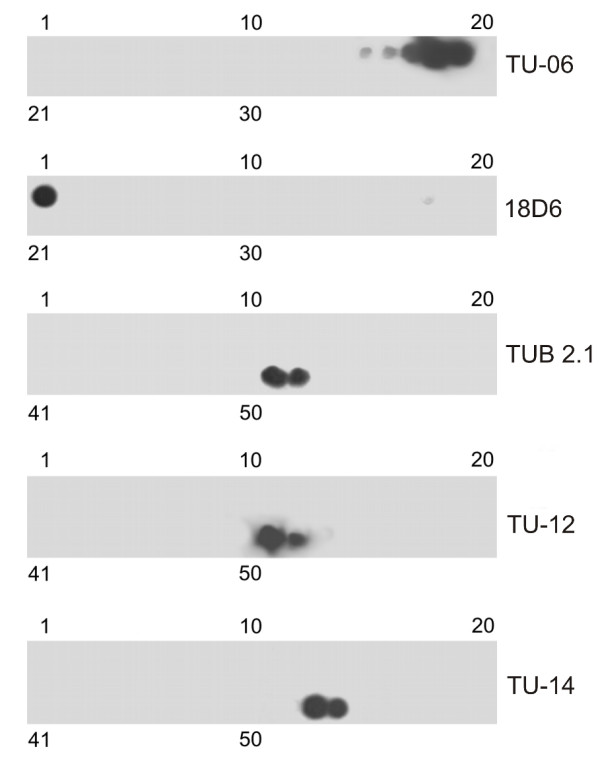
**Immunostaining of peptide scans covering *A. thaliana *β-tubulin 1 sequences with monoclonal antibodies**. Staining with antibodies 18D6 and TU-06 on peptide scans β1-180. Staining with antibodies TU-12, TUB 2.1 and TU-14 on peptide scans β171-447. Peptide scans were formed by immobilized linear 15-meric peptides with 5 amino acid overlaps. Numbers at the top and bottom denote peptide spots in the upper and lower row of the scan, respectively.

Peptide scans of immobilized overlapping peptides were used for a more accurate epitope location on *A. thaliana *β-tubulin. Two peptide scans were selected covering the regions β1-180 (34 linear 15-meric peptides with 5 amino acid overlaps) and β171-447 (54 linear 15-meric peptides with 5 amino acid overlaps) in β-tubulin 1 (TBB1). Results of immunostaining with the antibodies are shown in Figure 2. Using this approach the epitopes were located in the following β-tubulin regions: β1-10 (18D6), β81-95 (TU-06), β426-435 (TU-12), β426-435 (TUB 2.1) and β436-447 (TU-14). The comparison of epitope sequences, recognized by antibodies, with corresponding consensus sequences derived from all β-tubulin isotypes of *A. thaliana *is shown in Table 1. These data demonstrate that epitopes recognized by antibodies 18D6, TU-06 and TU-12/TUB 2.1 are highly conserved and are present in all β-tubulin isotypes. Less conserved is the C-terminal region, β436-447, where the epitope recognized by antibody TU-14 is located. Collectively taken, the data demonstrate that the identified β-tubulin epitopes are conserved and present in *A. thaliana *β-tubulins.

**Table 1:** Epitope location on *A. thaliana *β-tubulins*

Antibody		Region of epitope location
		1 10
18D6	TBB1	**M R E **I L H V Q G G
	Consensus	**M R E **I L H I Q G G
		81 95
TU-06	TBB1	**P Y G Q **I **F R P **D **N F V F G Q**
	Consensus	**P Y G Q **I **F R P **D **N F V F G Q**
		426 435
TU-12/TUB 2.1	TBB1	**Y Q D A T A D E E D**
	Consensus	**Y Q D A T A D E E G**
		436 447
TU-14	TBB1	**E Y D E E E E Q **- - **V Y E S**
	Consensus	**E Y E E E E E E E E E**

* *A. thaliana *consensus β-tubulin sequences were generated from *A. thaliana *sequences for TBB1 (P12411), TBB2 (P29512), TBB4 (P24636), TBB5 (P29513), TBB6 (P29514), TBB7 (P29515), TBB8 (P29516) and TBB9 (P29517) using Vector NTI Advance programme (InforMax, Bethesda, MD, USA). Swiss-Prot accession numbers are in parentheses. Identical amino acid positions are underlined. Amino acid residues exposed on inner or outer side of microtubule wall are in bold.

### Location of β-tubulin epitopes on a model of *A. thaliana *microtubule protofilaments and in cells

**Figure 3:**
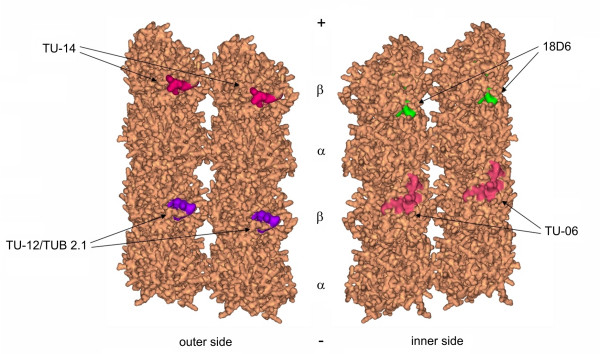
**Model of *A. thaliana *microtubule protofilaments with denoted positions of β-tubulin epitopes**. Amino acids exposed on surface of protofilaments are colored for epitopes recognized by monoclonal antibodies 18D6, TU-06, TU-12, TUB 2.1 and TU-14. Outer and inner sides of adjacent protofilaments are depicted. α and β denote positions of tubulin subunits in protofilaments; (+) and (-) mark the orientation of protofilaments.

The position of tubulin dimers in microtubule wall is known for brain tubulin [8,9]. Homology modeling of *A. thaliana *microtubules revealed that epitopes 18D6 and TU-06 are exposed to the lumen of microtubules, whereas epitopes for antibodies TU-12/TUB 2.1 and TU-14 are on the external side of microtubules exposed to cytoplasm. Exposures of the epitopes on *A. thaliana *microtubule protofilaments are depicted in Figure 3. Amino acids that were expected to be exposed on microtubular surface are highlighted in Table 1.

**Figure 4:**
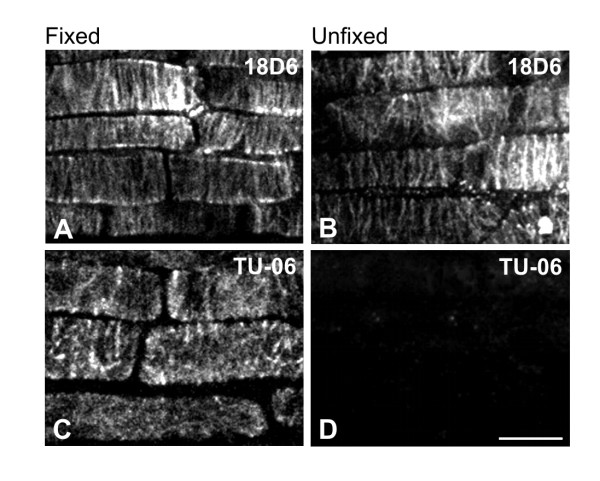
**Immunofluorescence staining of *A. thaliana *microtubules with antibodies to β-tubulin**. Fixed (A, C) or unfixed, detergent-extracted (B, D) preparations of primary root epidermal cells were prepared as described in Material and Methods and stained with monoclonal antibodies 18D6 (A-B) and TU-06 (C-D). Bar, 10 μm.

To verify the predicted location of epitopes on microtubules, immunofluorescence experiments were performed on fixed and unfixed detergent-extracted samples prepared from *A. thaliana *seedlings. All tested anti-β-tubulin antibodies stained microtubules in aldehyde-fixed *A. thaliana *seedlings. The antibodies decorated typical microtubule structures appearing during the cell cycle (interphase microtubules, preprophase band, mitotic spindle and phragmoplast). The antibodies stained microtubules along their whole length, and no differences among individual antibodies were detected. TU-06 failed to stain microtubules on unfixed detergent-extracted cells, in contrast to other antibodies that did stain all microtubule structures. Staining of interphase cortical microtubules in fixed and unfixed *A. thaliana *epidermal cells of primary roots under different fixation conditions is demonstrated for antibodies 18D6 and TU-06 (Figure 4A-D). The absence of reactivity with TU-06 could not be attributed to the lack of microtubules, since it was still possible to visualize microtubules by polyclonal anti-tubulin antibody (not shown). The reactivity of antibodies with *A. thaliana *microtubules are summarized in Table 2. These data demonstrate that epitopes TU-12/TUB 2.1 and TU-14, located in the C-terminal structural domain of β-tubulin and predicted to be on the microtubule surface, are indeed available for antibody staining in unfixed plant cells. On the other hand, TU-06 epitope exposed to the microtubule lumen was not accessible for antibody binding in unfixed microtubules, but fixation made the exposure of that epitope possible. Interestingly, the 18D6 epitope, located on the very N-terminal end of β-tubulin and also predicted to be exposed towards the microtubular lumen, was available for antibody binding in unfixed preparations.

**Table 2:** Reactivity of anti-β-tubulin antibodies with *A. thaliana *microtubules

Antibodies	Epitope location	Microtubules	
		
		Unfixed	Fixed
18D6	1-10	+	+
TU-06	81-95	+	-
TU-12/TUB2.1	426-435	+	+
TU-14	436-447	+	+

+, decoration of microtubules; -, no decoration of microtubules

### Exposure of phosphotyrosines on microtubules

The screening, by means of DS Visualizer program, of tyrosine residues exposed in *A. thaliana *microtubule model disclosed that only tyrosines 426 and 437 out of the 16 tyrosine residues present in consensus sequence of all β-tubulin isotypes were completely exposed on the outer side of microtubule surface. Besides, tyrosines at positions 439, 444, 445, 447 and 451, present only on some β-tubulin isotypes, were found to be part of the flexible C-terminal tail. Thus, they too are exposed on the microtubular external side (Additional file 1).

**Figure 5:**
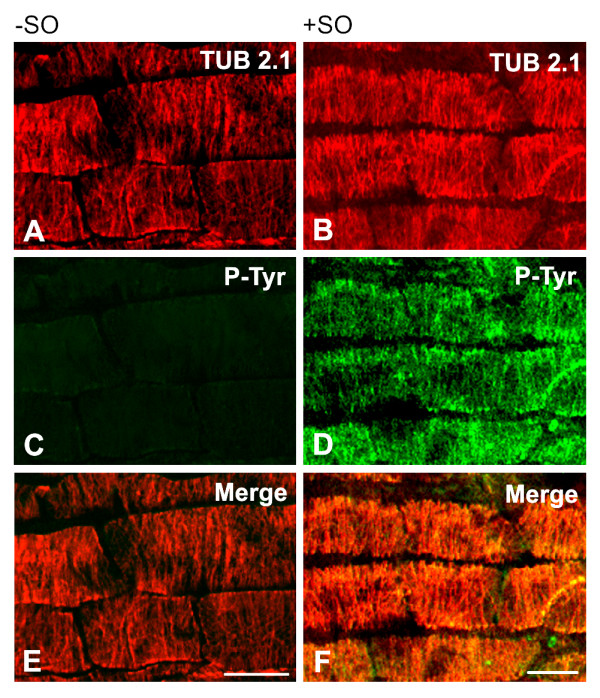
**Immunofluorescence double-label staining of *A. thaliana *unfixed microtubules with antibodies to β-tubulin and phosphotyrosine**. Preparations of primary root epidermal cells, preincubated without (A, C, E) or with (B, D, F) sodium orthovanadate (SO) to inhibit phosphatases were stained with monoclonal antibody TUB 2.1 to β-tubulin (A-B) and polyclonal anti-phosphotyrosine antibody P-Tyr (C-D). Figures A, C, E and B, D, F represent the same field. Superpositions (Merge) are shown in E and F. Bar, 10 μm.

To test whether or not plant microtubules can carry phosphorylation on tubulin tyrosine residues exposed on the outer side of microtubules, we have performed double-label staining experiments on unfixed detergent-extracted *A. thaliana *microtubules using polyclonal anti-phosphotyrosine antibody (P-Tyr) and monoclonal anti-β-tubulin antibody (TUB 2.1). Staining was also performed on seedlings pretreated for 40 min with 2.5 mM sodium orthovanadate. This compound is a potent inhibitor of protein tyrosine phosphatases, and proteins are therefore retained in their phosphorylated state. While the signal detected with anti-phosphotyrosine antibody in untreated samples was low (Figure 5C), staining was markedly enhanced if the cells were pretreated with sodium orthovanadate (Figure 5D). Double-label staining with anti-β-tubulin antibody confirmed that microtubules were preserved (Figures 5A and 5B), and phosphotyrosine labeling correlated with cortical microtubules in interphase cells (Figure 5F). Similar staining pattern with P-Tyr was observed when untreated or sodium orthovanadate-treated samples were fixed before immunostaining. Moreover, clear staining of mitotic spindles was also observed in fixed samples pretreated with sodium orthovanadate (not shown). These data suggest that plant microtubules can be modified by tyrosine phosphorylation and that tyrosine residues located in the C-terminal region of β-tubulin isotypes, exposed on microtubule surface, could be phosphorylated.

## Discussion

Homology modeling has been used for specification of some biophysical properties of tubulin isotypes [15], as well as for a rational design of microtubule inhibitors [16]. We made use of this approach to predict the exposure of β-tubulin epitopes and tyrosine residues on the surface of *A. thaliana *microtubules. Here, we report on three novel findings: (1) Identification of phylogenetically conserved β-tubulin epitopes recognized by well-established monoclonal antibodies. (2) Docking of epitopes into *A. thaliana *microtubule model and confirmation of predicted localization by staining of cellular microtubules. (3) Prediction of tyrosine exposure on the outer side of *A. thaliana *microtubule wall and experimental verification of microtubule phosphorylation on tyrosine in cells.

We confirmed the localization of epitopes for antibodies TU-06 (β81-95) [24] and TU-12 (β426-435) [25], previously determined on porcine brain tubulin, using *A. thaliana *overlapping β-tubulin peptides. We improved the accuracy of localization for epitopes recognized by antibodies 18D6 (β1-10), TUB 2.1 (β426-435) and TU-14 (β436-447). Out of this panel, antibodies TU-06, TU-12 and TUB2.1 are commercially available and are supplied by various vendors. Whereas the amino acid sequences in the regions β1-10 and β81-95 are highly homologous in all eight β-tubulin isotypes of *A. thaliana*, there are differences in the C-terminal regions of β-tubulin isotypes demonstrated by their amino acid alignment (Additional file 1). This indicates that the distribution of epitopes recognized by antibodies TU-12, TUB 2.1 and TU-14 might be restricted just to some isotypes. Identification of minimal peptides that bind to the antibodies is necessary before making final conclusions about the distribution of epitopes on individual β-tubulin isotypes. On the other hand, antibodies 18D6 and TU-06 represent general probes that ought to recognize all *A. thaliana *isotypes. As known posttranslational modifications of β-tubulin isotypes are located in the C-terminal domains [5,26], modifications should not impair the reactivity of these antibodies. 18D6 and TU-06 could be therefore useful for detection of all β-tubulin charge variants (isoforms) generated by combination of different β-tubulin isotypes and their posttranslational modifications.

Since the sequence alignment of porcine and *A. thaliana *β-tubulin isotypes revealed approximately 85% sequence identity, it is reasonable to assume that homology modeling based on known structure of bovine brain tubulin [6] combined with the high resolution model of microtubules [8,9] produces a model of *A. thaliana *microtubule protofilaments acceptable with a high degree of confidence. This enabled to position the β-tubulin epitopes into *A. thaliana *microtubule wall, and to localize those amino acid residues that are exposed on the inner or outer surface of microtubules.

While decoration of unfixed cellular microtubules was in good agreement with the predicted epitope docking for antibodies TU-06, TU-12, TUB 2.1 and TU-14, decoration of unfixed microtubules with antibody 18D6 was surprising. The 18D6 epitope in protofilament model was positioned in the microtubule interior facing the lumen, so one would expect that it will be obscured on unfixed microtubules. In addition, it has been demonstrated previously that microinjected 18D6 antibody did not decorate microtubules in animal CHO-K1 cells, if the cells were prior to fixation extracted with 0.5% Triton X-100 in microtubule stabilizing buffer [21]. When taxol was excluded during preparation of unfixed samples, staining of microtubules with 18D6 antibody was reduced (Additional file 2). An altering effect of taxol on the arrangement of tubulin protofilaments was reported [27,28]. We thus propose that the exposure of 18D6 epitope on unfixed *A. thaliana *microtubules might be caused by conformation changes generated by taxol used for stabilization of microtubules. Interestingly, only three out of the ten amino acids of the 18D6 epitope are exposed on the interior surface of microtubules (Table 1); this indicates that the N-terminal β-tubulin region is important for antibody binding to microtubules.

On the other hand, finding that epitopes 18D6 and TU-06, located on the inner site of microtubule wall, appeared after fixation of microtubules was no surprise. It is well documented that different fixation conditions influence the exposure of tubulin epitopes both in animal [29] and plant microtubules [17]. Since tubulin subunits have flexible domains [30], even "mild" fixation with 0.2% formaldehyde can affect the exposure of epitopes on microtubules [13]. It was suggested that changes in the conformation of tubulin by fixation influence the interaction between protofilaments, and can consequently lead to an exposure of epitopes in the lumen of microtubules, as was demonstrated for acetylated lysine α40 [13]. Conformation changes in protofilaments were reported after binding of the motor proteins to microtubules [31,32]. Our results strengthen the hypothesis that the way samples are prepared can substantially influence the results obtained in cells, and a good deal of caution must be used when predicting locations inferred from microtubule models.

Previously we have shown that plant tubulin is phosphorylated on tyrosine [18]; however, data on tyrosine phosphorylation of microtubules in plant cells are missing. Homology modeling in *A. thaliana *revealed that tyrosines in the C-terminal region of β-tubulin isotypes (behind β425) are exposed on the microtubule outer side and might therefore become likely targets for tyrosine kinases. In the case of α-tubulin isotypes, homology modeling disclosed that out of the total of 17 tyrosine residues only the C-terminal tyrosine α450 was completely exposed on the surface of microtubules (Additional file 1). To verify that tyrosines on polymerized microtubules are indeed phosphorylated, immunofluorescence experiments were carried out with unfixed cells. The results showed that the anti-phosphotyrosine antibody decorated microtubules. While the steady-state level of microtubule phosphorylation on tyrosine was low, phosphorylation of microtubules was clearly visible in cells pretreated with sodium orthovanadate, a factor which shifts the equilibrium to the phosphorylation state. Although one cannot exclude that the tyrosines partly buried in the microtubule wall are exposed to phosphorylation after binding of kinases to microtubules, it seems reasonable to assume that phosphorylation on tyrosines could occur in the C-terminal regions of polymerized tubulins. Since immunoblotting after 2D-PAGE separation of isolated *N. tabacum *tubulin clearly confirmed phosphorylation of both subunits [18], we suggest that β-tubulin as well as α-tubulin isotypes in *A. thaliana *microtubules might be phosphorylated. MAPs could also be phosphorylated on tyrosine in sodium orthovanadate-treated cells. However, MAPs are less abundant than tubulin in microtubule preparations isolated from plant extracts, and show usually punctuate localization in cortical *A. thaliana *microtubules [33]. In contrast, we have observed a uniform staining along the microtubules with anti-phosphotyrosine antibody in fixed cells. This indicates that anti-phosphotyrosine antibody might stain tubulin. However, further studies are necessary to confirm this assumption.

It is well established that the C-terminal domains of tubulins in polymerized microtubules can be posttranslationally modified [26] and bind MAPs [2]. The physiological significance of tyrosine phosphorylation of microtubules is unknown, but it can possibly modulate the binding of MAPs or motor proteins. In addition, it might modulate the function of microtubules in response to extracellular stimuli. Cellular microtubules can provide a matrix upon which the signaling molecules are arrayed [34]. Tyrosine phosphorylation of tubulin in microtubule polymer might affect the cell signaling by enhancing the Src-homology 2 (SH2) domain-mediated interaction between microtubules and signaling molecules. Syk-dependent phosphorylation of microtubules during activation of B-lymphocytes was described [35]. The Syk kinase belongs to protein tyrosine kinases of Syk/ZAP family, and tubulin is one of the proteins in animal cells identified as a likely substrate for Syk *in vivo *[36]. It is worth mentioning that ZAP-like kinases were identified by bioinformatics homology analysis in plant cells (Y.B. Blume unpublished result). Recent experiments with tyrosine kinase and phosphatase inhibitors identified tyrosine phosphorylation as an important factor in overall organization of microtubules in *A. thaliana *root cells [37].

## Conclusions

Using homology modeling we have generated an *Arabidopsis thaliana *microtubule protofilament model that served for the prediction of surface exposure of five β-tubulin epitopes, as well as tyrosine residues. Peptide scans newly disclosed the position of epitopes detected by antibodies 18D6 (β1-10), TUB2.1 (β426-435) and TU-14 (β436-445). Experimental verification of obtained results by immunofluorescence microscopy revealed that the exposure of epitopes was dependent on fixation. Moreover, homology modeling showed that only tyrosines located in the C-terminal region of β-tubulins (behind β425) are exposed on the microtubule outer side. Immunofluorescence microscopy confirmed tyrosine phosphorylation of microtubules in plant cells, and β-tubulins could thus be one of the targets for tyrosine kinases. Tyrosine phosphorylation of microtubules may affect their capability to interact with associated proteins in cells responding to extracellular stimuli. The data also demonstrate that however useful the homology modeling may be for the prediction of amino acid exposure on microtubule surface, it has to be corroborated by experimental verification.

## Methods

### Antibodies

The following mouse monoclonal antibodies to β-tubulin were used: TU-12 (IgM) recognizes an epitope located in the β426-435 region [25] of the C-terminal structural domain [38]; TU-14 (IgM) recognizes an epitope located in the isotype-defining region [19] of the C-terminal structural domain; TU-06 (IgM) recognizes an epitope located in the β81-95 region [24] of the N-terminal structural domain of β-tubulin [29]; TUB 2.1 (IgG1) (Sigma, Prague, Czech Republic) recognizes an epitope located in the C-terminal structural domain of β-tubulin [20]; 18D6 (IgG1) was raised against the N-terminal peptide of β-tubulin (β1-12) [21]. Absence of any cross-reactivity with α-tubulin has previously been confirmed for all anti-β-tubulin antibodies used in this study [17,21,39]. Microtubules were also visualized by affinity purified rabbit antibody to tubulin [38]. Mouse monoclonal antibody VI-01 (IgM) to vertebrate vimentin [40] was used as a negative control. Rabbit antibody to phosphotyrosine was from Upstate Biotechnology (Lake Placid, NY, USA). Anti-mouse antibody conjugated with horseradish peroxidase was purchased from Promega Biotech (Madison, WI, USA). Anti-mouse antibodies conjugated with fluorescein isothiocyanate (FITC) or indocarbocyanate (Cy3), and anti-rabbit antibody conjugated with FITC were obtained from Jackson Immunoresearch Laboratories (West Grove, PA, USA).

### Cells

Seedlings of *Arabidopsis thaliana *(Columbia 0 ecotype) were grown under sterile conditions on a half strength Murashige and Skoog (MS) medium containing vitamins (Duchefa, Haarlem, Netherlands), supplemented with 10 g/L sucrose and solidified with 4 g/L Gelrite (Duchefa) at pH 5.7. After overnight incubation at 4°C, the dishes were placed vertically in a growth chamber at 22°C in a 16/8 h (light/dark) photoperiod. Four-day-old seedlings were used for experiments.

### Protein preparation and epitope mapping

Microtubule protein from porcine brain was prepared by three temperature-dependent cycles of assembly and disassembly according to Shelanski [41]. Tubulin free of microtubule-associated proteins was obtained by phosphocellulose chromatography [42], and was stored in small aliquots in liquid nitrogen. To separate α- and β-tubulin subunits, tubulin was carboxyamidomethylated and the subunits were effectively separated by SDS-PAGE according to Laemmli using modifications in separation gel and electrode buffer composition [43]. Subunits were isolated from the gel by electroelution using Model 422 Electro-Eluter (Bio-Rad Laboratories, Richmond, CA) according to manufacturer's directions. Electroelution was performed in 50 mM ammonium bicarbonate containing 0.1% SDS. Eluted proteins were concentrated in Speed Vac (SAVANT Instruments, Farmingdale, NY).

Chemical proteolysis of β-tubulin was performed by dissolving the isolated protein in 75% formic acid and incubating the solution in the dark at 37°C for 24 h [22]. After dialysis against water using a dialysis membrane with MWCO 3,500 Daltons, samples were mixed with five-times concentrated SDS-sample buffer and analyzed by immunoblotting.

For epitope mapping, the synthetic overlapping peptides (15-meric peptides with 5 amino acid overlaps) were prepared by SPOT synthesis (Jerini Peptide Technologies, Berlin, Germany). Each spot carried approximately 5-nmol peptide covalently bound to the cellulose-β-alanine membrane. Peptide scans covered the sequences β1-180 in the N-terminal structural domain (34 peptides) and β171-447 in the C-terminal structural domain (53 peptides) of *A. thaliana *β-tubulin 1 (TBB1, accession number P12411 in the Swiss-Prot Sequence Database). Epitope mapping was performed according to the manufacturer's directions with chemiluminescent detection of bound antibodies.

### Preparation of cytoskeletons

Fixation of *A. thaliana *seedlings was adapted from Le *et al*. [44]. All incubation and washing steps were performed at room temperature. Briefly, whole seedlings of *A. thaliana *were fixed for 2 h in a freshly prepared mixture of 4% (w/v) paraformaldehyde and 0.5% (v/v) glutaraldehyde in PHEM buffer (60 mM PIPES-KOH, pH 7.0, containing 25 mM HEPES, 2 mM EGTA, 2 mM MgSO_4_) supplemented with 0.05% (v/v) Triton X-100 and 0.2 mM phenylmethane sulfonyl fluoride (PMSF). After rinsing in PHEM the seedlings were incubated in 0.1% (w/v) NaBH_4 _in PHEM for 15 min to reduce free aldehydes. Cell wall was digested for 20 min with an enzyme cocktail containing 0.1% (v/v) pectinase (Sigma) and 0.05% (w/v) pectolyase (Sigma) in PHEM. Seedlings were washed in PBS and incubated for 20 min in 1% (w/v) BSA in PBS to prevent unspecific binding of antibodies.

To prepare unfixed samples, *A. thaliana *seedlings were treated with the enzyme cocktail, washed twice for 10 min in 0.4 M glycerol in PHEM (glycerol/PHEM) and incubated for 15 min in the same buffer containing 1% (v/v) Triton X-100, 0.2 mM PMSF and 10 μM taxol (Sigma). In some cases, taxol was omitted from incubation buffer during preparation of unfixed samples. After washing in glycerol/PHEM, the cells were used for immunostaining.

For some experiments, seedlings were pretreated for 40 min at room temperature with 2.5 mM sodium orthovanadate (Sigma) before preparation of fixed or unfixed samples, in order to inhibit tyrosine phosphatases. 250 mM sodium orthovanadate stock solution in H_2_O was prepared immediately before use [37].

### Immunofluorescence microscopy

All antibody dilutions were made with 0.5% (w/v) BSA in PBS supplemented with 2 μM leupeptin (Sigma). Antibodies TU-06, TU-12 and TU-14 in the form of culture supernatants were diluted 1:50, antibodies TUB 2.1 and 18D6 were diluted 1:100. Fixed samples were incubated with primary antibodies for 2.5 h, washed 3 times for 5 min in PBS and incubated for 1.5 h with FITC-conjugated anti-mouse antibodies diluted 1:30. *A. thaliana *seedlings were incubated with antibodies in suspension and transferred before examination on glass coverslips with a small drop of PBS.

For experiments with unfixed cytoskeletons, seedlings of *A. thaliana *were incubated for 2.5 h with primary antibodies diluted in glycerol/PHEM. After washing off the unbound antibodies by glycerol/PHEM, samples were fixed for 1.5 h with 4% (w/v) paraformaldehyde in glycerol/PHEM. Samples were then washed with PHEM without glycerol and incubated for 1.5 h with secondary antibody as described above, followed by transfer on glass coverslips.

For double-label immunofluorescence examination of fixed or unfixed samples of *A. thaliana *seedlings, the rabbit anti-phosphotyrosine antibody P-Tyr (1:100) and the mouse monoclonal antibody TUB 2.1 (1:100) were applied simultaneously. FITC-conjugated anti-rabbit and Cy3-conjugated anti-mouse antibodies were diluted 1:100 and 1:500, respectively.

Preparations of epidermis of *A. thaliana *primary root were examined with confocal laser scanning microscope LSM 510 META (Carl Zeiss, Jena, Germany) with a Plan-Apochromat 63× oil-immersion objective. FITC emission was excited using the 488-nm ray of the argon laser, whereas Cy3 was exited using the 543-nm line of the helium/neon laser. Emission signals of FITC and Cy3 were separated by means of META system with, respectively, BP 505-530 and LP 560 filter.

### Gel electrophoresis and immunoblotting

Details on the one-dimensional SDS-polyacrylamide gel electrophoresis (SDS-PAGE), electrophoretic transfer of separated proteins onto nitrocellulose, and immunostaining procedure are described elsewhere [45]. SYPRO Ruby Protein Blot Stain (Invitrogen, Carlsbad, CA, USA) was used for the detection of proteins on nitrocellulose. The antibodies TU-06, TU-12 and TU-14 in the form of culture supernatants were diluted 1:5, TUB 2.1 and 18D6 were diluted, respectively, 1:2,000 and 1:10,000. Peroxidase-conjugated antibody was diluted 1:10,000. Bound antibodies were detected by SuperSignal WestPico Chemiluminescent reagents according to manufacturer's directions (Pierce, Rockford, IL, USA).

### Microtubule protofilament modeling

The spatial structure of αβ-tubulin heterodimers were reconstructed by the homology modeling method [46] using a previously described procedure [47]. For spatial model development, *A. thaliana *amino acid sequences corresponding to tubulin alpha-1 chain (P11139; Swiss-Prot Sequence Database) and tubulin beta-1 chain (P12411; Swiss-Prot Sequence Database) were applied. Preliminary geometry optimization of dimer spatial structure was calculated by the L-BFGS method [48] using the *mdrun *module of GROMACS software [49]. Spatial organization of tubulin protofilaments was computed using serial translation of the heterodimer along microtubule axis on a distance of 79.4 Å. Relative arrangement of protofilaments in microtubule was reconstructed by serial geometric transformations including protofilament axial rotation by 14 degrees and protofilament shift angularly of 10 degrees on a distance of 53 Å. Spatial structures of calculated longitudinal and lateral contact interfaces were refined to known data about amino acid residues which can be involved in forming the appropriate contacts [8,9]. Data visualization and analysis of spatial localization of antibody epitopes were done using the DS Visualizer 2.0 software (Accelrys Software Inc., Cambridge, United Kingdom).

## Authors' contributions

YB conceived the study, participated in homology modeling and helped in drafting the manuscript. AY performed the immunofluorescence experiments with inhibitors and critically evaluated the obtained data. YS prepared the cytoskeletons and participated in confocal microscopy. AN generated the microtubule protofilament model. TS carried out the epitope mapping and immunoblotting experiments. VS isolated the microtubule proteins and tubulins. PD participated in the design and coordination of this study, prepared the tubulin fragments and drafted the manuscript. All authors read and approved the final version of the manuscript.

## Supplementary Material

Additional file 1 **Supplementary Table 1S**. Alignment of carboxy-terminal domains of β- and α-tubulins in *A. thaliana*. Click here for file

Additional file 2 **Supplementary Figure 1S**. Effect of taxol on immunofluorescence staining of *A. thaliana *unfixed microtubules. Click here for file
