# Supplementary material for: Exposure of beta-tubulin regions defined by antibodies on an Arabidopsis thaliana microtubule protofilament model and in the cells
Source: BMC Plant Biol. 2010 Feb 18;10:29. doi: 10.1186/1471-2229-10-29 (PMC2844066; doi:10.1186/1471-2229-10-29)
Supplement: Additional file 1 — Supplementary Table 1S. Alignment of carboxy-terminal domains of β- and α-tubulins in A. thaliana. [file 1471-2229-10-29-S1.PDF]

Supplementary Table 1S - Alignment of carboxy-terminal domains of β- and α-tubulins in *A. thaliana*

Beta tubulins

TBB1_ARATH	(398)	W**Y**TGEGMDEMEFTEAESNMNDLVSE**Y**QQ**Y**QDATADEEDE**Y**DEEEEQ--V**Y**ES--
TBB2_ARATH	(397)	W**Y**TGEGMDEMEFTEAESNMNDLVSE**Y**QQ**Y**QDATADEEGD**Y**EDEEEGE**Y**QQEEE**Y**
TBB4_ARATH	(397)	W**Y**TGEGMDEMEFTEAESNMNDLVAE**Y**QQ**Y**QDATAGEE-E**Y**EEEEEEE**Y**ET-----
TBB5_ARATH	(398)	W**Y**TGEGMDEMEFTEAESNMNDLVAE**Y**QQ**Y**QDATADEEGE**Y**DVEEEEEGD**Y**ET--
TBB6_ARATH	(397)	W**Y**TGEGMDEMEFTEAESNMNDLVSE**Y**QQ**Y**QDATADDEGE**Y**EEDEDEEEILDHE-
TBB7_ARATH	(397)	W**Y**TGEGMDEMEFTEAESNMNDLVSE**Y**QQ**Y**QDATADEEGE**Y**EEEE-AE**Y**EQEET**Y**
TBB8_ARATH	(397)	W**Y**TGEGMDEMEFTEAESNMNDLVSE**Y**QQ**Y**QDATADEEEG**Y**E**Y**EEDEVEVQEEQ-
TBB9_ARATH	(397)	W**Y**TGEGMDEMEFTEAESNMNDLVAE**Y**QQ**Y**QDATVGEE-E**Y**EEDEEEEEA-----
Consensus	(398)	W**Y**TGEGMDEMEFTEAESNMNDLVSE**Y**QQ*YQDATADEEG*E***Y***EEEEEEEE E*
Epitopes		**TU-12/TUB2.1** **TU-14**

Alpha tubulins

TBA1_ARATH	(398)	M**Y**SKRAFVHW**Y**VGEGMEEGEFSEAREDLAALEKD**Y**EEVGGEGAEDDDEEGDE**Y**
TBA2_ARATH	(398)	M**Y**AKRAFVHW**Y**VGEGMEEGEFSEAREDLAALEKD**Y**EEVGAEGGDDEDDEGEE**Y**
TBA3_ARATH	(398)	M**Y**AKRAFVHW**Y**VGEGMEEGEFSEAREDLAALEKD**Y**EEVGAEGGDDEEDEGED**Y**
TBA6_ARATH	(398)	M**Y**AKRAFVHW**Y**VGEGMEEGEFSEAREDLAALEKD**Y**EEVGAEGGDDEDDEGEE**Y**
Consensus	(398)	M**Y**AKRAFVHW**Y**VGEGMEEGEFSEAREDLAALEKD**Y**EEVGAEGGDDEDDEGEE**Y**

Multiple alignment of tubulin isotypes (UniProtKB entry names are indicated) was performed by Vector NTI Advance programme (InforMax, Bethesda, MD, USA). Positions of tyrosine residues are in bold. The location of epitopes for antibodies TU-12/TUB 2.1 and TU-14 are in underlined italics and italics, respectively.
