# Supplementary material for: Exposure of beta-tubulin regions defined by antibodies on an Arabidopsis thaliana microtubule protofilament model and in the cells
Source: BMC Plant Biol. 2010 Feb 18;10:29. doi: 10.1186/1471-2229-10-29 (PMC2844066; doi:10.1186/1471-2229-10-29)
Supplement: Additional file 2 — Supplementary Figure 1S. Effect of taxol on immunofluorescence staining of A. thaliana unfixed microtubules. [file 1471-2229-10-29-S2.PDF]

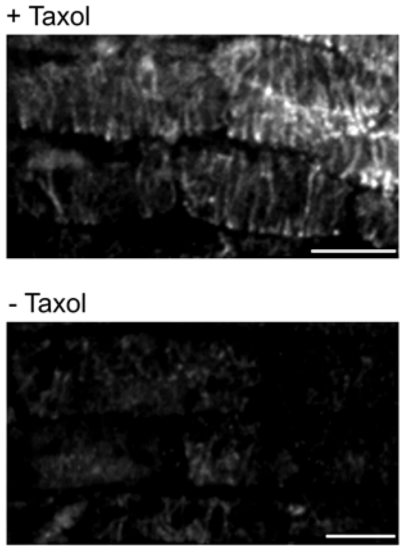

Supplementary Figure 1S – Effect of taxol on immunofluorescence staining of *A. thaliana* unfixed microtubules.

Unfixed, detergent-extracted epidermal cells of primary roots were prepared in the presence (+Taxol) or absence of taxol (-Taxol) and stained with monoclonal antibody 18D6. Bar, 10 μm.
